# Prevalence of Depression Among Adults With Diabetes Mellitus and the Relationship Between Improvement in Depressive Symptoms and Glycemic Control

**DOI:** 10.7759/cureus.48241

**Published:** 2023-11-03

**Authors:** Jessica L Saenz, Victor R Villarreal

**Affiliations:** 1 Family and Community Medicine, Oceania University of Medicine, Apia, WSM; 2 Family Medicine, Villarreal Medical Center, San Juan, USA

**Keywords:** comorbid diabetes and depression, diabetes and depression prevalence, type 2 diabetes and depression, depression and diabetes, diabetes and depression

## Abstract

Introduction: Depression is one of many comorbid conditions associated with diabetes. The rationale for this study is to examine the prevalence of depressive symptoms in adults with diabetes mellitus type II. Furthermore, the association between depressive symptoms and glycemic control will also be analyzed.

Materials and methods: A chart review of 59 diabetes mellitus type II patients from a family practice clinic in San Juan, Texas was performed. These patients were screened for depressive symptoms using the Patient Health Questionnaire-9 (PHQ-9) during their annual physical exam. Since many of these patients have been treated in this clinic for many years for their multiple comorbidities, it is possible to evaluate their responses to the PHQ-9 over consecutive years and compare them with their glycemic control using a HbA1c level. Data was evaluated by using biological parameters, such as age and gender, and the clinical parameter of a HbA1c level. Inferential statistics, such as prevalence, frequency, correlation, and *p-value, *were also used in analyzing the data.

Results: Depressive symptoms were analyzed using the PHQ-9. In 2016, 2017, and 2018, female diabetics were found to have a higher prevalence of depressive symptoms. When comparing diabetes and age, in 2016, those who were between the ages of 65 and 74 and 75 and older had a higher incidence of depressive symptoms. Furthermore, in 2017 and 2018, patients 75 and older also had a higher frequency of depressive symptoms. The controlled diabetic population was also found to have a greater rate of depressive symptoms. However, after careful analyzation, there was no significant relationship between glycemic control and depressive symptoms.

Conclusion: Many diabetics have comorbid depressive symptoms. Even though this study showed no relationship between depressive symptoms and glycemic control, the treatment of depressive symptoms in diabetics may help to prevent the multiple life altering complications that diabetes can cause.

## Introduction

Diabetes mellitus type II can be viewed as the consequence of a series of pathophysiologic changes, each of which makes the patient prone to subsequent disruption of normal glucose homeostasis [1]. In the United States, nearly 37.3 million individuals have some form of glucose intolerance or diabetes. Of these 37.3 million individuals, there are 28.7 million individuals already diagnosed with diabetes and 8.5 million who have undiagnosed diabetes. On the other hand, 122.4 million are considered to be prediabetic [2]. The long-term effects of uncontrolled diabetes are responsible for the majority of the morbidity and mortality [1]. Aside from some of the most well-known researched complications of diabetes, such as retinopathy, peripheral neuropathy, cardiovascular disease, and chronic kidney disease, diabetes is associated with psychiatric disorders, with depression being one of the most common [3]. More importantly, the severity of chronic complications is related to glycemic control [1].

About 15% of the general population will experience an episode of major depression at some point in their life [4]. According to one study, diabetic patients are more likely to experience depressive symptoms as compared to those who are not diabetic. Not only have 8.5-14% of type II diabetic patients suffered depressive symptoms at some point in their life, but they also have a lifetime risk of 11-32.5% [3,5].

Diagnostic criteria for major depressive disorders (MDDs) include depressed mood, anhedonia, and/or fatigue. More importantly, patients would need to have experienced at least one of these symptoms for at least two weeks in order to be diagnosed with an MDD [6].

Some studies suggest that the relationship between type II diabetes and depression coincides. They go on to infer that diabetes increases the risk of depression and on the contrary, depression increases one’s risk of developing diabetes [7]. Depressed diabetics are associated with an increased risk of experiencing severe medical complications, such as myocardial infarctions, vascular disease, and early mortality. As a result, these patients are understood to have a poorer overall health status and decreased adherence to self-care responsibilities, such as a proper diet and exercise regimen, as well as medication treatments. It has also been shown that medical costs associated with depressed diabetics are 4.5 times higher than those of patients with diabetes alone [5,7,8].

The current study was designed to investigate the prevalence of depressive symptoms in adults with type II diabetes mellitus and to analyze whether there is an association between depressive symptoms and glycemic control. Furthermore, the author hypothesizes that depressive symptoms are more prevalent in uncontrolled diabetic patients (HbA1c >7%).

## Materials and methods

In this retrospective study, 59 type II diabetic patients (26 female and 33 male) from a family practice clinic in San Juan, Texas were analyzed. Inclusion criteria were a diagnosis of diabetes mellitus II based on the American Diabetes Association (ADA) guidelines. Diabetic patients who have been diagnosed with cancer or other psychiatric conditions (e.g., schizophrenia, bipolar, etc.) in accordance with the DSM-V classification system were excluded from the study. 

During their annual exam, patients were screened for depressive symptoms using the Patient Health Questionnaire-9 (PHQ-9). Patient's scores ranged from 0 to 27, helping to screen for the symptoms of none to minimal depressive symptoms (score 0-4), mild depressive symptoms (score 5-9), moderate depressive symptoms (score 10-14), moderately severe symptoms (score 15-19), or severe depressive symptoms (score 20-27) [9]. Glycemic control was assessed by measuring HbA1c levels, which is a method for estimating glucose levels over the last three months. Patients with a HbA1c level greater than 6.5% were considered to be diabetic. Furthermore, if their level was greater than 7%, they were characterized as being uncontrolled diabetics. 

Over the span of three years, patient’s PHQ-9 scores and HbA1c levels were collected. The data were analyzed by using biological parameters, such as age and gender, and the clinical parameter of a HbA1c level. Inferential statistics, such as prevalence, frequency, correlation, and *p-value,* were also used in analyzing the data.

The study protocol was approved by the Oceania University of Medicine Institutional Review Board. Informed consent was obtained from the physician and anonymity was guaranteed. There was no cost associated with the study.

## Results

A chart review of 59 diabetes mellitus type II patients was analyzed over the span of three consecutive years. There were 26 female patients and 33 male patients. Furthermore, they ranged in age from 58 to 85 years with a mean age of 70.5 years and a standard deviation of 5.67 years. Patients were categorized into three age groups: 58 to 64 years of age (mean age of 59, 4 patients), 65 to 74 years of age (mean age of 69.3, 45 patients), and 75 years and older (mean age of 80.5, 10 patients). They were also grouped as either being a controlled diabetic (HbA1c < 7%) (or an uncontrolled diabetic (HbA1c > 7%).

Table 1 shows the overall prevalence of depressive symptoms based on the PHQ-9. As previously mentioned, patients are considered to have minimal depressive symptoms if their scores ranged from 1 to 4, mild depressive symptoms (score 5-9), moderate depressive symptoms (score 10-14), moderate severe depressive symptoms (score 15-19), or severe depressive symptoms (score 20-27) [9]. In 2016, 2017, and 2018, there were 59%, 53%, and 47% of patients who were considered to have some form of depressive symptoms, respectively.

**Table 1:** Overall prevalence of the major depression disorder (MDD) based on the PHQ-9 PHQ-9: Patient Health Questionnaire-9

2016	2017	2018
MDD	MDD	MDD
59%	53%	47%
(35)	(31)	(28)

Table 2 shows the frequency of depressive symptoms and its relation to sex, age, and blood glucose control. Only those patients who scored a 1 or higher were considered to have a positive PHQ-9 result and were included in this type of analysis. In 2016, 2017, and 2018, there were 61.54%, 61.54%, and 53.65% of female patients who were considered to have depressive symptoms, respectively. On the other hand, in 2016, 2017, and 2018, there were 60.61%, 45.45%, and 42.42% of male patients who were considered to have depressive symptoms, respectively.

**Table 2:** Prevalence of the major depression disorder (MDD) based on the PHQ-9 according to sex, age, and blood glucose control PHQ-9: Patient Health Questionnaire-9

	2016	2016	2017	2017	2018	2018
	MDD	MDD	MDD	MDD	MDD	MDD
	Positive	Negative	Positive	Negative	Positive	Negative
Female	61.54%	38.46%	61.54%	38.46%	53.65%	46.15%
	16	10	16	10	14	12
Male	60.61%	39.39%	45.45%	54.55%	42.42%	57.58%
	20	13	15	18	14	19
	2016	2016	2017	2017	2018	2018
	MDD	MDD	MDD	MDD	MDD	MDD
	Positive	Negative	Positive	Negative	Positive	Negative
58-64 years	50.00%	50.00%	50.00%	50.00%	50.00%	50.00%
	(2)	(2)	(2)	(2)	(2)	(2)
65-74 years	60.00%	40.00%	46.67%	46.67%	44.44%	48.89%
	(27)	(18)	(21)	(21)	(20)	(22)
> 75 years	60.00%	40.00%	80.00%	50.00%	60.00%	70.00%
	(6)	(4)	(8)	(5)	(6)	(7)
	2016	2016	2017	2017	2018	2018
	MDD	MDD	MDD	MDD	MDD	MDD
	Positive	Negative	Positive	Negative	Positive	Negative
Controlled (< 7%)	60.00%	40.00%	57.89%	42.11%	47.62%	52.38%
	(24)	(16)	(22)	(16)	(20)	(22)
Uncontrolled (>7%)	57.89%	42.11%	42.86%	57.14%	47.06%	52.94%
	(11)	(8)	(9)	(12)	(8)	(9)

When analyzing depressive symptoms in relation to age, in 2016, 60% of patients in the age group of 65-74 and 75 and older were found to have depressive symptoms. Moreover, in 2017, 80% of patients who were 75 and older showed a higher inclination of having depressive symptoms as compared to the year of 2018, where 60% of patients in the same age group had a positive PHQ-9 result. Furthermore, when analyzing depressive symptoms in relation to glucose control, in 2016, 2017, and 2018, the prevalence of depressive symptoms was greater in the controlled diabetic population. In 2016, 60% of controlled diabetics had a positive PHQ-9 result. In 2017 and 2018, there were 57.89% and 47.62% of controlled diabetics that had some form of depressive symptoms, respectively.

Table 3 shows the overall correlation between HbA1c and PHQ-9 scores for the years of 2016, 2017, and 2018. In all three years, there was a weak and negative correlation (r = -0.151, r = -0.074, and r = -0.015 respectively).

**Table 3:** Overall correlation between HbA1c and PHQ-9 PHQ-9: Patient Health Questionnaire-9

2016	2017	2018
Correlation	Correlation	Correlation
-0.151	-0.074	-0.015

Table 4 shows the correlation and p-value between HbA1c and PHQ-9 scores according to sex in 2016, 2017, and 2018. For all three years, there was not a significant relationship between these two values for both males and females. For males, in 2016, there was a negative and insignificant relationship between their HbA1c level and PHQ-9 result, r (31) = -0.221, p > 0.05. Furthermore, in 2017 and 2018, there was a positive, but insignificant relationship between their HbA1c level and PHQ-9 result, r (31) = 0.026, p > 0.05. For females, in 2016 and 2017, there was a negative and insignificant relationship between their HbA1c level and PHQ-9 result, r (26) = -0.133, p > 0.05 and r (26) = -0.212, p > 0.05, respectively. Furthermore, in 2018, there was a positive but insignificant relationship between their HbA1c level and PHQ-9 result, r (26) = 0.085, p > 0.05.

**Table 4:** Correlation and p-value between HbA1c and PHQ-9 scores according to sex PHQ-9: Patient Health Questionnaire-9

	2016	2017	2018
	Correlation	Correlation	Correlation
Male	-0.221	0.026	0.026
	(0.216)	(0.888)	(0.887)
	2016	2017	2018
	Correlation	Correlation	Correlation
Female	-0.133	-0.212	0.085
	(0.518)	(0.298)	(0.679)

Table 5 shows the correlation and p-value between HbA1c and PHQ-9 scores according to blood glucose control. For all three years, there was not a significant relationship between these 2 values for both controlled diabetics and uncontrolled diabetics. For controlled diabetics (HbA1c < 7%), in 2016, 2017, and 2018, there was a positive but insignificant relationship between their HbA1c level and PHQ-9 result, r (40) = 0.022, p > 0.05, r (38) = 0.028, p > 0.05, and r (42) = 0.251, p > 0.05, respectively. For all three years, patients that were considered to be uncontrolled diabetics (HbA1c > 7%), also had a positive, but insignificant relationship between their HbA1c level and PHQ-9 result, r (19) = 0.407, p > 0.05, r (21) = 0.207, p > 0.05, r (17) = 0.273, p > 0.05. Even though there were several positive correlations between HbA1c and PHQ-9 scores according to blood glucose control, the results were not sufficient to prove that a statistical significance existed.

**Table 5:** Correlation and p-value between HbA1c and PHQ-9 scores according to blood glucose control PHQ-9: Patient Health Questionnaire-9

	2016	2017	2018
Controlled (< 7%)	0.022	0.028	0.251
	(0.892)	(0.867)	(0.108)
Uncontrolled (>7%)	0.407	0.207	0.273
	(0.083)	(0.368)	(0.290)

## Discussion

Approximately 5% of the general United States population have been diagnosed with having the clinical syndrome known as major depression [10,11]. Furthermore, the incidence of major depression among diabetics has been investigated over the years and an association with the female gender has been previously confirmed in multiple research studies [3,10,12-15]. This study analyzed data over the span of three consecutive years. The overall prevalence of depressive symptoms among diabetics in this study ranged between 47% and 59%. More importantly, when comparing gender and depressive symptoms among diabetics, in 2016, 2017, and 2018, the frequency of depressive symptoms was greater in female diabetics. 

Earlier studies also showed a relationship between age and depression in diabetics. In one study, researchers showed that diabetics in the age group of 31-59 had a higher tendency of having major depression [3]. Yet, this study showed that in 2016, diabetic patients in the age group of 65-74 and 75 and older showed a higher propensity for having depressive symptoms. Moreover, patients with the age of 75 and older also had a higher incidence of depressive symptoms in the years of 2017 and 2018. When analyzing depressive symptoms in relation to glucose control, in all three years, 2016, 2017, and 2018, the prevalence of depressive symptoms was greater in the controlled diabetic population.

Another statistic that was analyzed was the correlation and p-value between HbA1c and PHQ-9 scores according to sex and glycemic control in 2016, 2017, and 2018. Results failed to show that a statistically significant relationship existed between glycemic control and depressive symptoms. However, previous studies did find a statistically significant relationship between depression incidence and glycemic control [3,8,10]. One reason as to why these studies’ results may differ from this study is that a larger sample was analyzed. The results of previous studies help to support the theory that as glycemic control improved, patients lowered their risk of being depressed [3].

According to data from the National Health Interview Study, diabetic patients who have comorbid depression are 7 times more likely to become disabled when compared to others who have either disease alone. Furthermore, those who are depressed diabetics have a 2.3 times increased risk of early mortality when compared to nondepressed diabetics [5,16-18].

In summary, depression can be catastrophic in patients, especially diabetics. Some challenges, other than a psychiatric disability that it can cause, include an impairment in work or social activities, family difficulties, and a financial hindrance [5,8,19]. Even after patients have been screened for depression, there still are other challenges that healthcare providers face when trying to provide the appropriate treatment for these patients. Some obstacles include patient refusal of treatment for their depression, insufficient numbers of providers, untrained staff, cultural barriers, and limited access to mental health treatment centers [5,20-22].

Future studies are indicated to determine whether treatment of comorbid depression in diabetics, especially uncontrolled diabetics, would not only help reduce hyperglycemia, but in turn, can help to decrease the multiple complications and life-altering effects that diabetes can cause, such as retinopathy, peripheral neuropathy, cardiovascular disease, and chronic kidney disease [23].

Limitations

This study has several limitations. One limitation is that the questions on the PHQ-9 were read/asked to the patient by a medical assistant rather than the patient reading the PHQ-9 for themselves, thus, possibly creating a response bias. Furthermore, many of the patients speak Spanish and the PHQ-9 forms are written in English. As a result, the medical assistant would assist in translating to the patient. Another limitation was that a control group was not included in this study. The patient’s history of illegal drug use, alcohol use, or their socioeconomic status was not considered. One strength was that the sample collected from this one primary health care clinic comprised a good representation of adults in primary health care throughout the lower Rio Grande Valley in South Texas. Still another strength was the availability of the laboratory data HbA1c.

## Conclusions

In summary, the results of this study of adults with type II diabetes showed that depressive symptoms are more common among females. Even though this study failed to show that a significant relationship between glycemic control and depressive symptoms existed, other studies did show that diabetics with good glycemic control were less likely to be depressed than those with poor glycemic controls. More importantly, additional research is needed in order to determine if the treatment of depression in diabetics may help to prevent the multiple life-altering complications that diabetes can cause.
